# Complete Genome Sequence of Rahel, a C1 Cluster Mycobacteriophage

**DOI:** 10.1128/MRA.01071-20

**Published:** 2020-11-05

**Authors:** Fernando E. Nieto-Fernandez, Christos Noutsos, Jillian Nissen, Yara Abdelsalam, Jessica Ackloo, Navpreet Banger, Hason Chan, Tarana Chittineedi, Isabel Duplessy, Mark Dyce, Daesha Garrison, Jaime Gonzalez, Sandra John, Imanjot Kahlon, Tania Kumar, Adwoa Lewis, Karthik Madhira, Rivka Mullokandova, Nellie Pirzadeh, Iman Raja, Kevin Ram, Ravi Ramdhari, Rita Reddy, Briana S. Saed, Phalan Smith, Steeve Sproul, Jane Thomas, Avia Yossefi, Jorge Morales

**Affiliations:** a Department of Biological Sciences, SUNY Old Westbury, Old Westbury, New York, USA; b The City College of New York, New York, New York, USA; Queens College

## Abstract

Rahel is a lytic *Myoviridae* bacteriophage that infects Mycobacterium smegmatis mc^2^155. It has 1,555,955 bp and 64.7% G+C content. Rahel has a circularly permuted genome with 270 genes, 53 of them of known function, 33 tRNAs, and 1 transfer-messenger RNA (tmRNA). Only five genes are coded on the reverse strand.

## ANNOUNCEMENT

**FIG 1:**
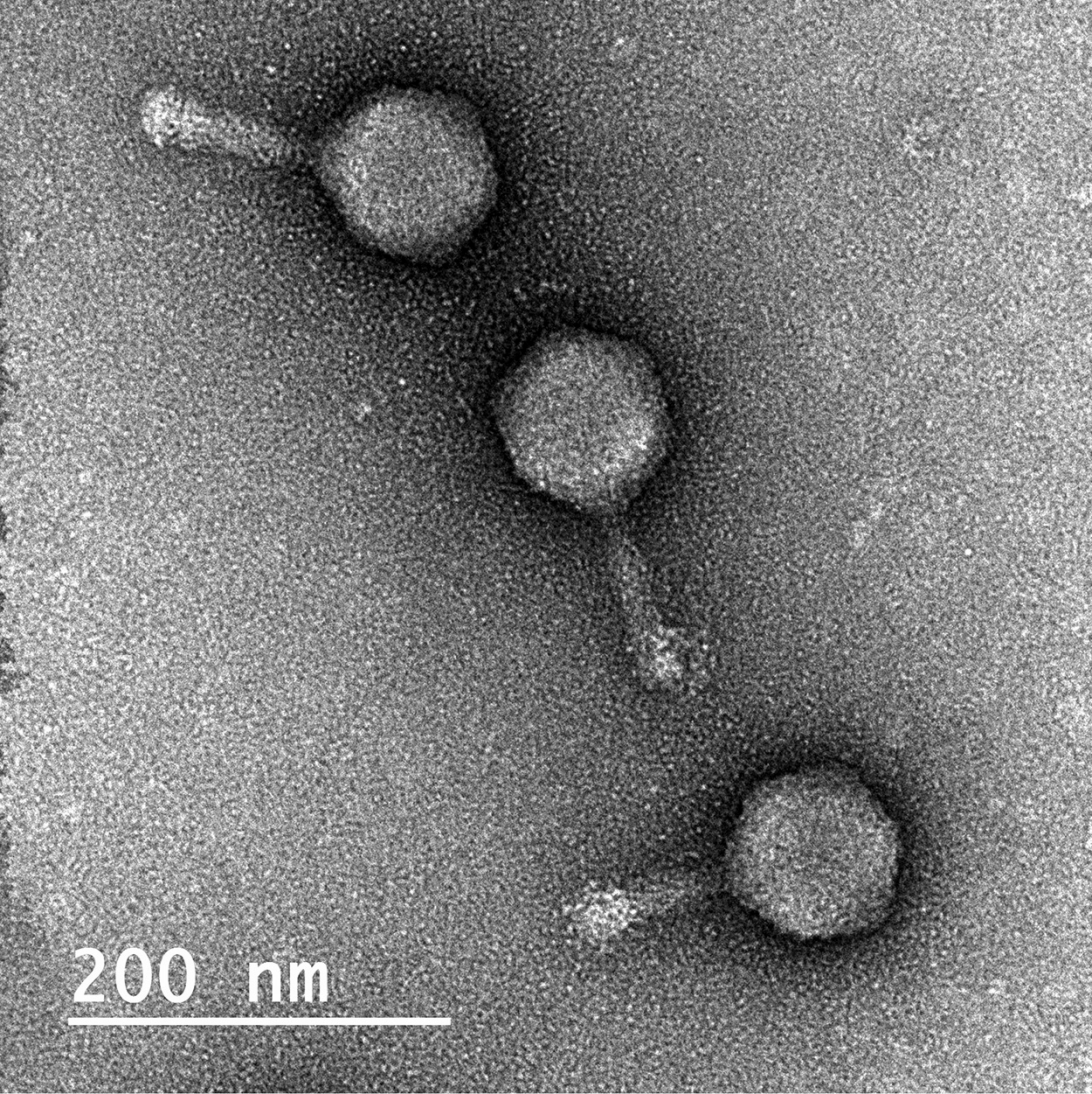
TEM of Rahel mounted on Formvar-coated copper grids stained with uranyl acetate and imaged with a JEOL JEM-2100 microscope. Rahel’s virions are 178 nm in total length, with an isometric head and tail of 89 nm (×50,000 magnification).

Rahel was isolated as part of the Science Education Alliance-Phage Hunters Advancing Genomics and Evolutionary Science (SEA-PHAGES) at SUNY Old Westbury. Rahel was isolated in Hicksville, Nassau County, New York (lat 40.7679, long −73.5405). It is a C1 subcluster lytic phage that infects Mycobacterium smegmatis mc^2^155. Clusters are groups of phages with sequence similarity over 50% of their genome (1). It was isolated by enrichment and two cycles of purification and amplification in 7H9 top agar at 37°C (2). For transmission electron microscopy (TEM), phages were collected from a high-titer lysate by high-speed centrifugation, mounted on carbon-stabilized, Formvar-coated copper TEM grids stained with uranyl acetate, and imaged with a JEOL JEM-2100 TEM (2). Rahel is a *Myoviridae* of 178 nm total length with an isometric head and tail of 89 nm (Fig. 1). DNA was extracted from a high-titer lysate using a Promega DNA Wizard kit with a modified protocol (2). Genomic DNA libraries were generated using an Ultra II FS kit (New England Biolabs [NEB], Ipswich, MA) with dual-indexed barcoding. Pooled libraries were run on an Illumina MiSeq system at the University of Pittsburgh, which yielded at least 547,000 single-end 150-base reads. Read coverage depth was 471× without Sanger finishing reactions. The reads were assembled using Newbler and Consed (3). A full genome sequence was present, as the contigs on both ends overlapped and the genome circularized. Rahel was annotated using DNA Master (http://cobamide2.bio.pitt.edu/computer.htm) and the gene prediction tools GLIMMER v3.0 (4), GeneMark v2.5 (5), and Starterator v1.1 to determine gene start sites. tRNA and transfer-messenger RNA (tmRNA) predictions were made using ARAGORN v1.2.38 (6) and tRNAscan-SE v3.0 (7). Functional assignments were made using BLAST v2.9 (8), HHpred (9), and Phamerator (10). Default settings were used for all programs.

Rahel has 155,955 bp, 64.7% G+C content, 270 open reading frames (ORFs), 33 tRNAs, 1 tmRNA, 53 genes of known function, and 182 hypothetical proteins (67.6%); only five ORFs are coded on the reverse strand. Rahel has a circularly permuted genome, as all of the reads assembled into a large contig and there was no large buildup of reads or coverage variation at the ends (2). When linearized, the coding region of gene 270 is disrupted. Structural genes include genes 99 (the major capsid protein) and 100 (the capsid decoration protein), genes 126 to 144 (the tail structural proteins, including the tail assembly chaperone), and genes 129 and 130. Rahel has a tail sheath protein gene, 126, and two baseplate wedge protein genes, 138 and 139, but a tail tube protein was not identified. Genes involved in nucleotide metabolism and DNA replication are found on the right end of the genome from gene 147 on to the end. In Rahel, the lysis system is encoded by genes 252 and 254, lysin A and B, respectively, followed by a terminase, gene 255. We did not find a holin gene as part the lysis system.

### Data availability.

The complete sequence for Rahel’s genome can be found in GenBank under accession number MK359348 and SRA accession number SRX9162484.
